# Reconstructing the hepatocellular carcinoma microenvironment: the current status and challenges of 3D culture technology

**DOI:** 10.1007/s12672-025-02290-z

**Published:** 2025-04-10

**Authors:** Ting Xiong, Kai Wang

**Affiliations:** 1 Division of Hepato-Biliary-Pancreatic Surgery, Department of General Surgery, The Second Affiliated Hospital, Jiangxi Medical College, Nanchang University, Nanchang, China; 2 Jiangxi Province Engineering Research Center of Hepatobiliary Disease, The Second Affiliated Hospital, Jiangxi Medical College, Nanchang University, Nanchang, China; 3 The MOE Basic Research and Innovation Center for the Targeted Therapeutics of Solid Tumors, The Second Affiliated Hospital, Jiangxi Medical College, Nanchang University, Nanchang, China; 4 Jiangxi Provincial Clinical Research Center for General Surgery Disease, The Second Affiliated Hospital, Jiangxi Medical College, Nanchang University, Nanchang, China

**Keywords:** 3D culture technology, Hepatocellular carcinoma, Tumor microenvironment, Cell culture model

## Abstract

Hepatocellular carcinoma (HCC), with high incidence and mortality rates among digestive system diseases, has become a focal point for researchers. However, the more we learn about HCC, the more apparent it becomes that our understanding is still superficial. The successes and failures of numerous studies underscore the urgent need for precision medicine in cancer treatment. A crucial aspect of preclinical research in precision medicine is the experimental model, particularly cell culture models. Among these, 3D cell culture models can effectively integrate and simulate the tumor microenvironment, closely reflecting the in vivo conditions of patients. This capability provides a solid theoretical foundation for personalized treatment approaches. In this review, we first outline the common in vitro 3D cell culture models and examine the essential elements within the tumor microenvironment, followed by insights into the current state and future developments of 3D in vitro cell models for HCC.

## Introduction

Liver cancer is one of the most prevalent malignant tumors of the digestive system, characterized by high incidence and mortality rates, posing a significant threat to global public health. Although epidemiological data indicate a slight decline in the long-term global trends of liver cancer incidence and mortality, the five-year relative survival rate remains alarmingly low at only 22% [1]. Hepatocellular carcinoma (HCC) is the most common type of liver cancer, accounting for approximately 85% of cases [2]. Therapeutic strategies for HCC encompass surgical resection, liver transplantation, ablative therapy, endovascular interventional therapies (such as transarterial chemoembolization [TACE], transarterial radioembolization [TARE], and selective internal radiation therapy [SIRT]), and systemic antitumor therapy [3]. Liver transplantation is considered the most effective treatment option for eligible patients, particularly those with early-stage HCC and underlying liver cirrhosis. Surgical resection is indicated only for patients with adequate hepatic functional reserve. For patients without fibrosis or cirrhosis, the residual liver volume must exceed 30% of the standard liver volume, whereas those with chronic liver disease, liver parenchymal damage, or cirrhosis require a residual liver volume of more than 40% [4]. Currently, systemic therapy for advanced HCC primarily involves immunotherapy combined with angiogenesis inhibitors, such as atezolizumab plus bevacizumab, which has demonstrated superior efficacy compared to sorafenib in clinical trials [5–9]. Despite significant progress, these therapies are often limited by severe adverse effects and emerging resistance, driven by tumor heterogeneity and variations in the microenvironment [10]. This calls for the development of personalized strategies to further optimize treatment outcomes.

Reliable in vitro tumor models are crucial tools for achieving patient-specific therapies, which aim to provide customized treatment strategies based on the unique characteristics of each patient's tumor. Although two-dimensional (2D) cell models are widely used in preclinical studies due to their ease of use and lower costs, recent research has shown that the results from 2D models often deviate significantly from clinical outcomes, particularly in drug development and personalized treatment [11–14]. In certain diseases, including cancer, the 2D cell model fails to adequately replicate disease conditions, as it overlooks a critical factor in disease progression—the changes in the cellular microenvironment. In cancer research, alterations in the microenvironment not only directly make the tumor surroundings more conducive to cancer cell growth but also indirectly lead to drug resistance and distant metastasis [15, 16]. Therefore, studying the tumor microenvironment is an indispensable component in cancer-related research. Compared to 2D cell models, 3D culture models, which incorporate cell–matrix interactions and co-culture systems, better reveal the interactions between cells and between cells and the matrix, providing researchers with a more comprehensive and multi-dimensional understanding of the disease [17–19].

**Fig. 1:**
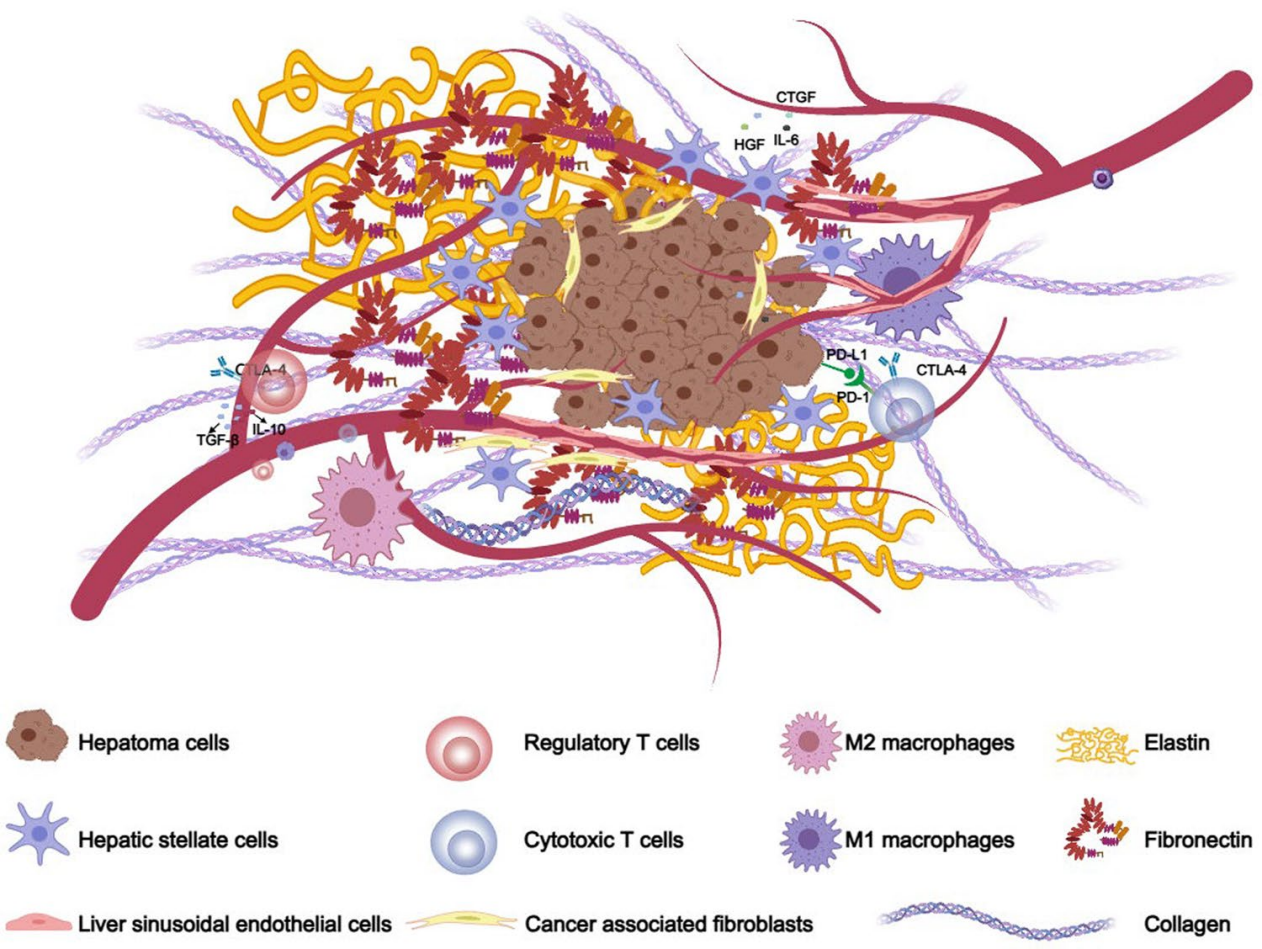
Hepatic tumor microenvironment schematic diagram. Hepatic stellate cells promote the proliferation and survival of cancer cells by secreting cytokines (e.g., IL-6) and growth factors (e.g., CTGF and HGF), and they enhance immune evasion through the upregulation of immune checkpoint molecules (e.g., PD-L1 and CTLA-4), thus achieving immune evasion and tolerance. Hepatic sinusoidal endothelial cells and cancer-associated fibroblasts, as structural components of the tumor microenvironment, participate in intercellular interactions and signal transduction while providing a physical barrier that hinders drug efficacy. Regulatory T cells assist cancer cells in evading immune surveillance by secreting immunosuppressive cytokines such as IL-10 and TGF-β, while cytotoxic T cells actively target and destroy tumor cells but can become less effective in the presence of immunosuppressive signals from Tregs and the tumor microenvironment. Cytotoxic T cells rely on pro-inflammatory cytokines to enhance their effector functions; however, regulatory mechanisms, including PD-1 engagement, often inhibit their activity. M1 macrophages exert anti-tumor effects by secreting pro-inflammatory factors, resisting tumor progression, whereas M2 macrophages inhibit intracellular inflammatory responses and promote tumor cell stemness through various mechanisms, thereby facilitating tumor progression. Various matricellular proteins are involved in the chemical and physical signaling pathways within the tumor, participating in the processes of tumor initiation and development. Created with MedPeer (medpeer.cn)

In the tumor microenvironment (TME) of HCC, aside from hepatocellular carcinoma cells, there are also various matrices and non-cancerous cells. The extracellular matrix (ECM) of a normal liver constitutes about 10% of the liver's total volume, and these matrix proteins work together with non-parenchymal cells to maintain hepatic homeostasis [20]. In HCC, collagens, elastin, and fibronectins are especially prominent components of the TME [21–24]. Besides these, the TME of HCC also includes various non-parenchymal cells, such as liver sinusoidal endothelial cells, hepatic stellate cells, fibroblasts, and immune-related cells [25–28] (Fig. 1). During the development and progression of HCC, these cells are not only victims of the altered TME but also active contributors to its construction [29]. Research focused on modulating the TME to hinder HCC progression is steadily increasing [30–33]. Therefore, there is a growing need to develop in vitro models that can accurately reflect the HCC TME. This review will primarily discuss the latest advances in 3D in vitro models of the HCC TME, analyze and compare the strengths and limitations of each model, and explore their applications, aiming to provide more references and suggestions for in vitro model development in HCC research.

### 3D cell culture models

As cells exist as three-dimensional structures, interactions between them should not be limited to a single interface. The 3D culture model is an improvement over the traditional 2D model, utilizing current technologies and resources to closely mimic the in vivo environment, thereby making research results more clinically relevant. In a 3D culture model, various cells and extracellular matrix components can be incorporated, and the cells themselves can also be genetically modified. Conducting multiple gene modifications in animal models is often cost-prohibitive and risky, as gene-edited mice are typically expensive, and multiple genetic alterations can lead to lethal outcomes. However, 3D cell culture models, combined with CRISPR-Cas9 technology, enable high-throughput screening of disease-causing genes [34–37].

Currently, various approaches have been employed to construct 3D HCC models. Among these, spheroid models are widely utilized due to their simplicity, low cost, and high reproducibility. These models can be generated using hanging drop techniques or low-adhesion plate methods, with HepG2 and Huh7 cell lines being commonly used. However, spheroid models are limited by their inability to adequately replicate cell–matrix and cell–cell interactions. In contrast, scaffold-based models, which utilize materials such as gelatin-alginate hydrogels, provide a more accurate structural representation of the tumor microenvironment. Patient-derived organoid models, on the other hand, offer higher fidelity in recapitulating tumor heterogeneity and genetic characteristics. However, these models are less accessible and require complex protocols to establish. Three-dimensional bioprinting technology further allows for precise control over cell placement, ECM composition, and physical properties. Although this method is expensive, it holds significant potential for developing complex and highly reproducible models. Co-culture systems, which integrate stromal and immune cells, enhance the disease relevance of 3D HCC models by replicating the TME more accurately. However, such systems may pose challenges for reproducibility due to their added complexity. Overall, standardization of tissue or cell sources, culture conditions, scaffold composition, and experimental protocols remains critical to achieving reproducible results in laboratory settings.

**Fig. 2:**
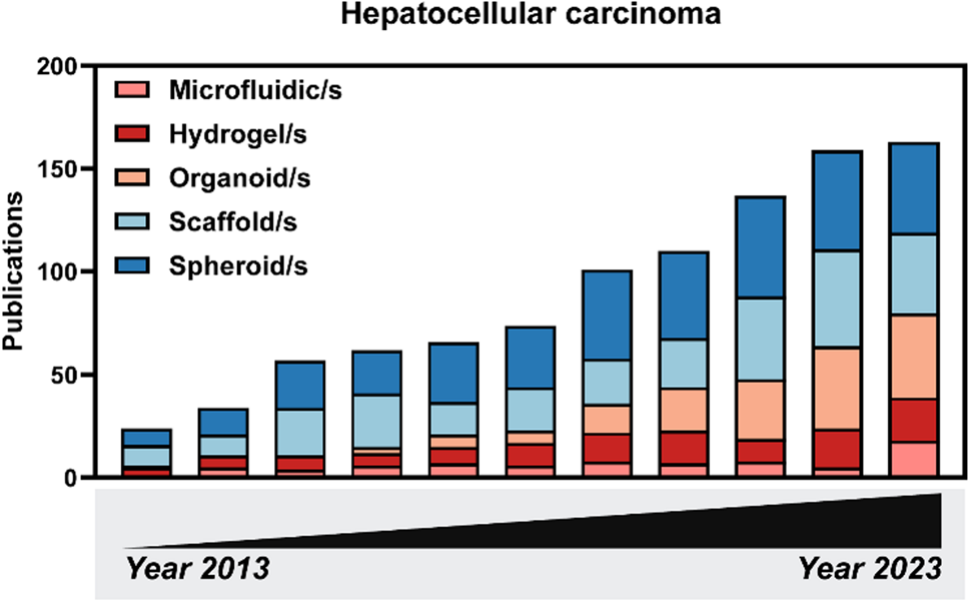
The number of publications each year regarding 3D cell culture methods in HCC research (PubMed search updated until September 10, 2024). A literature search was conducted using PubMed by combining the terms "microfluidics," "scaffolds," "3D cell culture," "organoids," and "spheroids" with "Hepatocellular carcinoma" in the title or abstract

We conducted a literature review on the application of 3D cell culture models in HCC and found that among the five types of 3D HCC cell models, spheroid models accounted for the largest proportion at 35.5%, followed by scaffold models (28.2%) and organoid models (16.3%). Lastly, hydrogel models (12.5%) and microfluidic models (7.6%) represented smaller portions (Fig. 2). Additionally, the chart indicates that research on hepatocellular carcinoma organoid models has been increasing annually since 2016 and may potentially surpass other 3D cell culture models in the future.

### Organoid and spheroid models

As a burgeoning 3D culture model, organoids have garnered significant attention from researchers [38, 39]. To standardize and clarify the term "organoid," over 60 international experts reached a consensus, defining organoids as three-dimensional structures derived from (pluripotent) stem cells, progenitor cells, and/or differentiated cells [40]. These structures self-organize through cell–cell and cell–matrix interactions, replicating various aspects of natural tissue structure and function in vitro [41]. Liver organoids can be categorized into cancer organoids and non-cancer organoids. Cancer organoids are often derived from patient tumor tissues or sources like ascites, while non-cancer organoids are primarily derived from pluripotent stem cells or primary hepatocytes [42].

Due to the diversity of subtypes and mutations in cancer cells, the effectiveness of targeted therapies can vary widely [43, 44]. Although a small number of mutations differing from the original tumor were identified in HCC organoids derived from tumor biopsies (accounting for a median of 15% and 12% of mutations present in HCC organoids, respectively, with most not occurring in bona fide cancer genes), they largely retained the genetic alterations and mutational characteristics observed in their original HCC. Moreover, two pathology experts with specialization in liver pathology conducted histological analyses and diagnostic evaluations of the original biopsy and its tumor organoids on paraffin-embedded sections. Notably, the HCC organoids preserved the growth pattern and differentiation grade of their originating primary tumors. Similar to the original tumor, the HCC organoids demonstrated a solid growth pattern with Edmondson grade III differentiation or pseudo-glandular formation [45]. However, the participation of more cells is required to simulate the original tumor TME. To better reflect the heterogeneity of the original tumor and replicate its TME, the design should comprehensively evaluate the pathological and molecular subtypes of the tumor, utilizing high-throughput screening and single-cell sequencing technologies to analyze tumor heterogeneity. The cancer cells in the model are ideally derived from multiple regions of the patient’s original tumor, and various cell types should be introduced to mimic the tumor microenvironment. By combining patient-derived organoids and 3D bioprinting technologies, the distribution of cells and the matrix can be precisely controlled to create complex tumor models, while also accounting for culture conditions such as cytokines and oxygen concentration. In addition to the drug resistance factors inherent to cancer cells themselves, changes in the surrounding tumor microenvironment can also impede the effectiveness of drugs. For example, Liu et al. successfully developed a 3D co-culture system combining liver cancer organoids with cancer-associated fibroblasts (CAFs) [46]. Their research revealed that the addition of CAFs led to the failure of several anti-cancer drugs, including sorafenib, underscoring the significant role CAFs play in therapeutic resistance in liver cancer. Cho et al. further developed a multicellular HCC organoid (MCHO) model by co-culturing various stromal cells with HCC cells. Their study, which involved delivering verteporfin to the MCHO model, revealed that the activation of YAP/TAZ impaired the drug penetration of verteporfin. This research not only confirms the significant role of the tumor microenvironment in cancer cell drug resistance but also elucidates the involvement of the YAP/TAZ signaling pathway in tumor drug resistance [47]. Patient-derived organoids (PDOs) have demonstrated promising results in clinical trials, with their drug sensitivity closely mirroring that of actual patients [48]. Rao et al. constructed a PDO from a 60-year-old cholangiocarcinoma patient and evaluated six chemotherapy regimens, with the PDO showing the highest sensitivity to the combined chemotherapy of irinotecan and cisplatin. Consequently, postoperative adjuvant chemotherapy with irinotecan and cisplatin was administered. After six cycles of chemotherapy, CA-199 levels returned to normal, and postoperative CT showed no lesion enlargement, indicating good clinical efficacy [49]. Similarly, Mo et al. reported that the drug sensitivity of PDOs from colorectal cancer with liver metastases showed results similar to patients' progression-free survival [50].

Organoid models have become adept at capturing key immune features of the TME and replicating individual biological changes and immune responses [38]. Given their ability to mimic complex biological processes, organoids have emerged as valuable tools for applications such as high-throughput drug screening. However, despite their potential, there are significant challenges associated with optimizing organoid culture systems for such purposes, which primarily involve three key aspects. Standardization of Culture Conditions: Organoid culture demands precise control over variables such as medium composition, temperature, and humidity. Additionally, organoids derived from different sources may require tailored culture conditions. While robotic systems can efficiently manage mechanical operations in batches, establishing standardized protocols for these batch processes remains essential. High-Throughput Data Processing and Analysis: Traditional organoid culture methods are typically manual, leading to inefficiencies and higher error rates. To enable high-throughput screening, many automated workflows and batch analysis tools have been developed. However, significant advancements are still needed in image analysis. Research by Oishi et al. highlighted two primary limitations: first, the time-intensive nature of acquiring images using confocal microscopy; second, the reliance on machine learning for automated 3D image quantification in batch analysis, which involves reclassifying LTL^+^ objects to accurately represent 3D structures. This additional step can introduce bias into the outcomes of large-scale screenings, necessitating further refinement of current technologies and analytical methods [51]. Stability and Reproducibility of Organoids: Over prolonged culture periods, organoids might undergo variations that compromise the stability and reproducibility of experimental results [52]. Moreover, organoids cannot fully replicate the complexity of the in vivo tumor microenvironment. As a result, organoid culture techniques and strategies continue to evolve, calling for the development of more innovative technologies to support high-throughput applications. Organoid models serve as potential patient-specific personalized models. With the introduction of multicellular components, tumor cells within organoids increasingly resemble those in the original tumor. Although current organoid models cannot fully represent the characteristics of the original tumor, the developmental trajectory of organoids aligns with the goals of personalized precision medicine.

Spheroids are cell aggregates formed by the simple clustering of cells. The suspension droplet culture method is often employed, wherein tumor cells are inoculated into a culture medium, allowing the cells to aggregate under the influence of gravity, thus forming a tumor spheroid within each droplet. These tumor spheroids can generate gradients of oxygen and nutrients inside the sphere, accompanied by a necrotic core, effectively simulating the hypoxic environment typically found in the center of in vivo tumors [53]. Tumor spheroids are widely used in cellular experiments to explore various functions of tumor cells. To meet the demands of high-throughput drug screening, microplate arrays are utilized for the efficient expansion of tumor spheroids. However, oxygen supply in such environments is often insufficient, resulting in suboptimal spheroid viability. To address this issue, He et al. introduced an oxygen-permeable micro-well device based on polydimethylsiloxane (PDMS) to generate highly viable and functional HCC spheroids, which can be used for high-throughput drug screening research [54]. In addition, when the spheroids are combined with the scaffolds, tumor cells proliferate in the tumor extracellular matrix, and these scaffolds can capture key features of the tumor microenvironment; the strategy of combining both is more effective in reflecting the true environment of the tumor compared to spheroids without scaffolds [55, 56].

Although tumor spheres are convenient in tumor research and can effectively reflect the tumor microenvironment, their lifespan is often short. In contrast, organoid models exhibit better biological similarity and meet the requirements for more precise and complex medical treatment, with a generally longer maintenance period compared to tumor spheres. Although the current cost of organoid research is still relatively high, as organoid research continues to increase in the future and with the development of interdisciplinary studies, the cost of organoid research may gradually decrease.

### Microfluidic culture system

Microfluidics refers to fluid systems with lengths in the sub-millimeter range, where surface tension and capillary forces are the primary forces at play. Microfluidics utilizes these forces at the microscopic scale to transport microfluids, filter various analytes, and convert multiphase fluids into monodispersed droplets [57]. Due to the initial unevenness of flux microfluidics in the first few minutes, the initial droplets are often unsuitable for analysis, leading to waste of samples and reagents. To address this, Zhai et al. invented a microfluidic chip that uses digital microfluidics to control droplets, preventing the loss of precious samples. Furthermore, in double-blind drug screening experiments, the results showed that a positive drug successfully delayed the recurrence of metastatic tumors in one patient, demonstrating its great potential in precision medicine [58]. In the study of tumor microenvironment, microfluidics allows for the co-culture of various cells and research involving microfluidic organoids. Microfluidic technology significantly saves time costs and enables high-throughput drug screening and therapy. By co-culturing collagen with four major liver-specific cell types (hepatocytes derived from HCC, hepatic stellate cells, Kupffer-like macrophages, and endothelial cells) and combining microfluidic technology, Özkan et al. constructed a novel vascularized hepatocellular carcinoma chip, which can be used to evaluate the vascular transport regulation and efficacy of embolization and systemic treatment methods during cancer progression [59]. Meanwhile, Zou et al. developed a liver cancer micro-engineering organ-on-a-chip by co-culturing patient-derived organoids with mesenchymal hepatocytes and peripheral blood mononuclear cells, thereby reconstructing the tumor immune microenvironment and precisely conducting drug sensitivity screening, reducing the time to just one week [60]. Despite the increasing enthusiasm for constructing tumor microenvironments using microfluidic systems for research on precision medicine in oncology, we must address issues such as complexity and stability. Microfluidic technology is characterized by a high level of complexity, with the design and fabrication of microfluidic chips requiring highly specialized techniques, which increases the complexity and cost of experiments. Moreover, standardization in microfluidics remains challenging due to the diversity and complexity of microfluidic systems, making standardization and large-scale production difficult to achieve. Some microfluidic materials may exhibit cytotoxicity, potentially compromising the reliability of experimental results. In addition, the massive amount of data generated in tandem with high-throughput analysis poses significant challenges for researchers, demanding higher levels of data processing and analysis capabilities. To accelerate the development of this technology, the integration of biomedicine, clinical medicine, and engineering will facilitate a more rapid and efficient advancement.

### Scaffold and hydrogel culture system

The application of 3D scaffolds in cancer research models demonstrates significant advantages, as these scaffolds can more accurately replicate the complexity of the liver cancer microenvironment, including the composition of extracellular matrix, mechanical properties, and their effects on cellular behavior. Various scaffolds have been employed in the study of drug resistance in liver cancer. Turtoi et al. developed the HA3P50 scaffold by cross-linking hyaluronic acid and poly (methyl vinyl ether-alt-maleic acid), providing a more physiologically relevant environment for the culture of HepG2 cells. For the first time, the functional performance of HepG2 cells in a 3D environment was examined in detail, including the production and metabolic recovery of albumin and bile acids, along with a systematic analysis of the changes in signaling pathways before and after cisplatin treatment [61].

Most research models on tumor microenvironments focus on cell culture media and multicellular co-culture strategies, while high-throughput screening studies on ECM have been limited. Ryoo et al. developed a model of a 3D heterogeneous microenvironment using protein-labeled polyethylene glycol microgels with variable stiffness, leveraging their combinatorial potential to evaluate hematopoietic stem cell (HSC) niche remodeling and metabolic activity within a high-throughput ECM screening system. This platform can be employed to identify the ECM composition/stiffness conditions that lead to optimal drug responses in HSCs, with the potential for future incorporation of patient-specific samples. Additionally, the injectability of microgels allows for the possibility of selecting ECM that is suitable for cellular interaction, to be co-injected with drugs at the tumor site following high-throughput ECM screening, thereby enhancing cell-drug interactions and promoting the cytotoxic effects of the drugs on tumors [62].

Angiogenesis plays a crucial role in the occurrence and progression of tumors, and even in tumor resistance, anti-angiogenic therapy remains an indispensable component [63, 64]. Lim et al. constructed a hydrogel system to establish a co-culture model that simulates and characterizes the interactions between HCC, endothelial cells, and the immune microenvironment in vitro [65]. Similarly, Wang et al. developed a 3D endothelialized liver tumor micro-tissue model based on multicellular aggregates of human liver cancer cells and human umbilical vein endothelial cells co-cultured in porous microbeads of poly(lactic-co-glycolic acid) (PLGA PM) [66]. Furthermore, this model also supports the inclusion of other cell types, such as fibroblasts (L929) and HepG2 cells, for co-culturing. Both models take into account the influence of vascular factors on the tumor microenvironment, providing more options for a more refined in vitro liver cancer model. The advantages of scaffold and hydrogel culture systems lie in their ability to simulate the acellular matrix components within the liver cancer microenvironment, particularly in reflecting the rigidity and adhesiveness of the direct contact between cells and the matrix. By integrating multicellular co-culture, these systems can recreate the complex intercellular interactions that occur during tumor growth, including critical phenomena such as vascular invasion, ultimately enabling a more accurate simulation of in vivo liver cancer models. However, the shortcomings of these systems must also be carefully considered; their complexity and lack of standardization may lead to uncontrolled variations in experimental results. Additionally, simulating dynamic environments, such as fluid shear forces, remains a challenge. Recently, 3D bioprinting technology has shown promising results in the construction of in vitro models. The Yilei Mao team successfully established a 3D HCC model using 3D bioprinting technology with primary human liver cancer cells [67]. The expression of HCC-specific biomarkers (alpha-fetoprotein, AFP) was stably preserved in patient-derived 3D-printed hepatocellular carcinoma models (3DP-HCCs), showing a similar expression pattern to that of the original HCC specimens. Across all six patients, a high level of single nucleotide variant (SNV) consistency was observed between each 3DP-HCC and its initially matched HCC tissue. Similarly, analysis of exonic variant proportions in each pair confirmed that the SNVs and insertions/deletions in the original HCC tissues were well retained during long-term culture. Each 3DP-HCC model maintained the mutation profiles of its initially matched HCC tissue during long-term culture (e.g., ASXL1, ATM, TP53, CSMD3, PKHD1). Furthermore, RNA-seq results demonstrated that the gene expression profiles of the original HCC tissues were stably preserved in the 3DP-HCCs during long-term culture. Recent advances in scaffold materials have focused on better replicating the unique mechanical, biochemical, and dynamic properties of the liver tumor TME. For instance, novel hydrogel-based scaffolds with variable stiffness gradients effectively mimic the spatial heterogeneity of ECM stiffness in liver tumors, providing insights into tumor cell invasion mechanisms [68]. Additionally, smart scaffolds responsive to external stimuli, such as pH or enzymatic activity, create dynamic environments that reflect in vivo conditions, aiding in a deeper understanding of drug resistance mechanisms in liver tumors. Furthermore, 3D bioprinting technology has emerged as a powerful tool for constructing highly organized liver tumor models. Unlike traditional scaffolds, bioprinted models offer unprecedented control over the spatial distribution of cells, ECM-like materials, and vascular networks, leading to more physiologically relevant interactions within the TME [69, 70]. However, limitations such as printing resolution, material biocompatibility, and scalability remain challenges that future research must address. Overall, integrating multifunctional scaffolds with advanced cell culture systems represents a promising approach for establishing robust liver tumor models for drug screening and mechanistic studies.

### Non tumor cellular components in the TME of HCC

Non-tumor cells in hepatocellular carcinoma mainly include hepatic stellate cells, cancer-associated fibroblasts, endothelial cells, and immune cells. These cells play an indispensable role in tumor phenotypes such as tumor cell proliferation, migration, and drug resistance. Investigating the crosstalk between these cells and tumor cells in 3D cell models will provide more therapeutic options for cancer treatment.

### Hepatic stellate cells (HSCs)

HSCs account for approximately 5–10% of the total hepatocyte population in the liver [71]. In their quiescent state, HSCs serve as the primary cells for storing vitamin A, while also secreting growth factors that promote normal cellular turnover and regulating the hepatic vasculature [72]. However, under the stimulation of liver cell-damaging factors such as inflammation, viral infections, or alcohol, HSCs become activated and transdifferentiate into activated myofibroblast-like HSCs, producing significant amounts of extracellular matrix to form scar tissue for liver repair. The application of single-cell sequencing technology and spatial analysis technology has provided a deeper understanding of HSCs, revealing that HSCs are not merely in a quiescent state and an activated state. Recent studies have identified the zonal distribution of two static HSC subpopulations based on the expression of the Ngfr and Adamtsl2 genes, referred to as “portal vein-associated HSCs (pahsc)” and “central vein-associated HSCs (cahsc)”, revealing the different patterns and types of liver injury can determine the recruitment and distribution of quiescent hepatic stellate cells (HSCs) [73–75]. Activated HSCs can also transdifferentiate into CAFs, supporting tumor growth, metastasis, and cancer cell stemness, while mediating immune suppression and drug resistance through the secretion of a set of factors and nutrients [76].

In HCC, HSCs promote the progression of cancer cells from multiple aspects. HSCs facilitate the proliferation and survival of cancer cells by secreting cytokines and growth factors, which also exert immunoregulatory effects by inhibiting the activity of cytotoxic T cells and promoting macrophage polarization, thereby achieving immune evasion and tolerance [77–79]. Research has also revealed that HSCs can enhance tumor drug resistance by influencing the distribution and metabolism of drugs, as well as altering the proliferation status of tumor cells. HSCs are also a major driving factor in the scarring of chronic liver disease. Recent studies have identified aging HSCs in chronic liver disease, which play a pro-inflammatory role in the progression of metabolic dysfunction-associated fatty liver disease, rather than promoting proliferation, ultimately leading to damage in hepatocytes [80]. Furthermore, HSCs can alter liver cancer cell metabolism by secreting extracellular vesicles that encapsulate hexokinase 1 (HK1), resulting in a shift towards accelerated glycolysis, thereby facilitating the progression of HCC [81]. Therefore, research on HSCs in HCC represents an essential aspect in understanding this malignancy. Shen et al. studied the crosstalk between activated HSC and HCC cells using a microfluidic platform to simulate physiological structures and precise spatiotemporal control. Combined with cytokine arrays and RNA sequencing analysis, they demonstrated that the iron-binding protein Lipocalin-2 (LCN-2) is a key regulatory factor in the tumor microenvironment, and targeting LCN-2 shows strong antitumor effects [82]. Additionally, as previously mentioned, the multicellular HCC organoid model also includes HSCs [47, 59].

### Cancer-associated fibroblasts (CAFs)

CAFs, as indicated by their name, are closely associated with cancer. CAFs can differentiate from resident fibroblasts or stellate cells in response to growth factor stimulation. In tumor tissues, they can also arise from bone marrow mesenchymal stem cells and endothelial cells [83]. Numerous studies have reported their various pro-cancer mechanisms in the TME. For example, CAF-derived CCL5 upregulates zinc finger E-box binding protein 1 (ZEB1) by inhibiting the ubiquitination and degradation of hypoxia-inducible factor 1 alpha (HIF1α), thereby inducing epithelial-mesenchymal transition (EMT) [84]. CD36 CAFs derived from HSCs mediate macrophage migration inhibitory factor (MIF) expression dependent on the lipid peroxidation/p38/CEBPs axis, recruiting CD33 myeloid-derived suppressor cells (MDSC) and facilitating the progression of HCC in vivo [85]. CAFs also promote the consolidation of the tumor extracellular matrix by secreting large amounts of collagen and fibronectin, thereby inhibiting the recruitment of immune cells to tumor cells and hindering the penetration of anti-tumor drugs.

By constructing a co-culture model of CAFs and organoids, the effects of CAFs on tumor progression can be explored in vitro. Liu et al. found that a 1:1 co-culture of organoids and CAFs significantly promoted the size of the organoid diameter. Notably, the organoids and CAFs used in the co-culture system were not derived from the same individual, which may introduce some bias in the results [46]. Dong et al. utilized alginate and gelatin to cross-link into hydrogel capsules and co-cultured them with patient-derived tumor organoids [86]. By analyzing the TME within the culture environment, they detected non-cellular components including liver tumor cells, CAFs, endothelial cells, and hepatocyte growth factor. Unfortunately, the authors did not assess other cellular components in the TME such as immune cells. Further preclinical evidence is needed to demonstrate the advantages of this model.

### Liver sinusoidal endothelial cells (LSECs)

LSECs are specialized endothelial cells arranged in the liver sinusoids, playing a crucial role in maintaining hepatic homeostasis and immune surveillance. Due to their large intercellular spaces and discontinuous basement membrane, they can effectively interact with immune cells and macromolecules in the blood. At the same time, in the context of liver cancer, the construction of the TME involving LSECs is also further influenced by capillarization [87–90]. LSECs promotes intratumoral angiogenesis in liver cancer by secreting VEGF, which further exacerbates liver fibrosis and tumor progression. Notably, the profibrotic effects of LSECs occur even earlier than activated HSCs [91, 92]. Furthermore, LSECs is also involved in immune regulation within the HCC microenvironment. Sharma et al. employed scRNA sequencing to extensively characterize the cellular landscape of the human liver from development to disease, revealing that endothelial cell oncogenic reprogramming drives immunosuppressive macrophages in hepatocellular carcinoma, uncovering previously unexplored tumor-fetal reprogramming within the tumor ecosystem [87]. Consequently, targeting vascularization in HCC treatment is an important aspect.

In clinical therapy, Eisenbrey et al. described a method combining ultrasound-triggered microbubble (MB) destruction with transarterial radioembolization (TARE) to enhance hepatocellular carcinoma treatment, showing improved therapeutic response compared to conventional radioembolization, with no changes in vital signs or liver function [93]. However, the study had a limited sample size of 28 patients, necessitating further evidence to validate the method's feasibility. At the cellular biology level, research found that sustained anti-angiogenic therapy increases tumor hypoxia levels, leading to elevated expression of HIF-1α, consequently resulting in resistance of HCC to anti-angiogenic therapy [89, 94]. Therefore, more studies are needed on the molecular pathways involving LSECs in HCC. Song et al. demonstrated that the multicellular tumor spheroid model (MCTS), constructed by comparing monolayer cultures, tumor spheroids, and patient-derived cells, including human hepatic stellate cells, human fibroblasts, and human umbilical vein endothelial cells, is the optimal method for screening and optimizing treatment for each HCC patient [95]. This model was validated solely from the perspective of cell resistance, and further evidence is required to corroborate the similarity of the MCTS-HCC model to the in vivo tumor microenvironment of personalized treatment for HCC involving additional phenotypes and clinical therapeutic evidence. Poddar et al. focused on endothelial cell research in liver cancer, constructing a three-dimensional microfluidic co-culture system containing endothelial cells and HCC cells to explore crosstalk between endothelial and cancer cells [96]. This chip features one inlet and three independent outlets, allowing for effective monoculture and co-culture. The controllable and replicable environment of the chip enables studies involving co-culturing with other cell types as well. Despite extensive research on endothelial cells, no studies have integrated the spatial distribution of LSECs in the liver, and the application of decellularized scaffolds may provide assistance.

### Immune cells

Immunotherapy is a critical step in the treatment of HCC; however, due to the presence of tumor heterogeneity, the efficacy of immunotherapy varies among individuals. Therefore, personalized precision medicine treatment should not be overlooked in this regard. Despite the complexity of the immune landscape within the tumor microenvironment, which has resulted in the absence of robust models that can fully simulate the immune microenvironment of solid tumors, there is good news: organoids have shown advantages in reconstructing the immune microenvironment [97–99]. By co-culturing immune cells with patient-derived tumor organoid models, the TME in patients can be simulated. Additionally, PDOs grown at the air–liquid interface exhibit an immune stroma similar to that of the original tumor, without the addition of exogenous immune cells. PDOs contain macrophages expressing CD14^+^ or CD68^+^ at varying levels. Flow cytometry analysis of PDOs has identified the presence of CD8 + (cytotoxic T cells, Tc) and CD4 + (helper T cells, Th) T cells, B cells, natural killer (NK) cells, and natural killer T (NKT) cells, as well as infiltrating CD3^+^ T cells expressing the immune checkpoint receptor programmed cell death protein-1 (PD-1). This model provides a new platform for studying the regulatory mechanisms of immune cell function, such as the impact of the PD-1/PD-L1 pathway on T cell activity, as well as the potential mechanisms underlying resistance to immunotherapy [97]. Immune cells can originate from tumor-infiltrating lymphocytes or peripheral blood immune cells. Here, we primarily focus on tumor-associated macrophages (TAMs) and T cells as two types of immune cells studied in these models with immune cells sourced from tumor-infiltrating lymphocytes or peripheral blood immune cells. Here, we primarily discuss the study of tumor-associated macrophages and T cells as two types of immune cells within these models.

### TAMs

TAMs are among the most abundant immune cells in the tumor microenvironment and play a critical role in inflammation regulation. Based on their diverse functions and phenotypic characteristics exhibited under different cytokine stimuli, macrophages are currently classified into M1 and M2 subtypes. M1 macrophages, known as pro-inflammatory macrophages, serve to kill tumor cells in the tumor stroma. However, research has also found some rare pro-cancer mechanisms of M1 macrophages, which activate the expression of PD-L1 in liver cancer cells by secreting IL-1β [100]. It has been shown that in the tumor microenvironment, M1 and M2 macrophages do not exist independently, with M2 macrophages being the predominant type [101]. M2 macrophages, also known as anti-inflammatory macrophages, play an immunosuppressive role in the microenvironment. They can promote the stemness of HCC through the secretion of various cytokines and exosome vesicles. Rapid tumor proliferation leads to local inadequate oxygen supply, and local hypoxia enhances the pro-angiogenic and immunosuppressive abilities of M2 macrophages [102, 103]. The construction time of organoid models derived from patients is often lengthy, however, adding mesenchymal stem cells (MSCs) or fibroblasts that can generate tumor stroma during culture can significantly shorten the time required for PDOs establishment. Additionally, incorporating peripheral blood mononuclear cells into the culture system allows for the differentiation of TAMs during co-culture. Under the presence of IL-2 (2000 IU/ml) and supplemented with MSC-conditioned medium, there is a slight inhibition of T cell proliferation, thereby creating an MSC-PDO-PBMC micro-engineered HCC chip organoid that can simulate an immunosuppressive tumor microenvironment, demonstrating substantial potential in precision medicine and immunotherapy [60].

### T cells

In the TME, T cells primarily include regulatory T cells and cytotoxic T cells, with regulatory T cells being the majority. Under normal physiological conditions, regulatory T cells participate in intracellular immune regulation and prevent the occurrence of autoimmune diseases. However, in the TME, an excess of regulatory T cells can inhibit immune cells from recognizing and killing tumor cells, leading to immune escape of tumor cells. In immunotherapy, the PD-1 receptor pathway and CTLA-4 pathway are two classical targets [104, 105]. The upregulation of PD-L1 on cancer cells and immune cells in the TME leads to the inactivation of cytotoxic T cells, as well as reduced T cell proliferation and diminished immune capability. Additionally, CTLA-4 on T cells binds to CD80 (B7-1) on antigen-presenting cells, producing negative immune effects; the combined signals from these two immune checkpoints have a synergistic effect against tumors [106]. However, the current studies on T cells in the TME predominantly utilize 2D cell models and mouse models, with limited application of 3D cell models. Voabil et al. developed and used a patient-derived tumor fragment platform to analyze the early immune response of human tumor tissue to PD-1 blockers [107]. The authors observed that the ability of immune cells to be reactivated in vitro could predict clinical responses, and they identified tumor-resident T cells as a key component of this immune response. Moreover, through a combined analysis of baseline characteristics and immune response capabilities, the authors discovered a new infiltrative tumor subtype lacking response capability to PD-1 blockade. This research provides evidence for tumor immune reactivation, offering more technical and theoretical support for subsequent exploration of tertiary lymphoid structures in tumors.

### ECM components in the TME of HCC

In the microenvironment of HCC, in addition to hepatic tumor cells and other non-tumor cell components, there are also infiltrating ECM components from non-tumor cells. The matrix components provide adhesion for tumor cells and facilitate cell proliferation. Furthermore, in the context of drug resistance in liver cancer, the matrix has been found to counteract the targeting ability of drugs towards tumor cells. ECM may promote liver cancer metastasis through the following mechanisms. First, changes in the components of the ECM (such as collagen, fibronectin, and laminin) can bind to integrin receptors and activate downstream signaling pathways (e.g., PI3K/AKT and FAK/Src), thereby enhancing the migratory and invasive properties of tumor cells. Second, ECM remodeling can induce epithelial-mesenchymal transition (EMT) through specific signaling pathways (e.g., TGF-β and Wnt/β-catenin), causing tumor cells to lose polarity and acquire migratory and invasive capabilities. Additionally, specific ECM proteins (e.g., fibronectin) and matrix cleavage products can promote angiogenesis, increasing the likelihood of tumor cells entering the bloodstream and creating conditions for distant metastasis. Recent studies have also revealed that the matrix components play a crucial role in immune regulation [108–111]. This information suggests that the influence of the extracellular matrix on tumor progression is much more significant than we previously imagined.

### Collagen

Collagen acts as a cell adhesive in various tumors and is the main structural protein of ECM. The secretion of collagen in HCC mainly comes from hepatic stellate cells, fibroblasts, and tumor associated fibroblasts. The process of liver fibrosis is closely related to the excessive accumulation of collagen fibers, especially type I collagen [112]. Type I collagen can stimulate EMT in liver cancer cells, promoting YAP overactivation and enhancing the stemness of HCC cells through its corresponding receptor, discoid protein domain receptor 1 (DDR1) [113, 114].

In HCC research, many models have considered the potential effects of collagen in the TME, utilizing collagen matrices as scaffolds or inducing collagen generation during the culture process. One study coupled porcine liver-derived collagen (COL) with dialdehyde groups of partially oxidized alginate (OA) to prepare an in vitro TME-simulating matrix, OA-COL, in which the cells exhibited enhanced tumor cell stemness [115]. Another study prepared an RGD (Arg-Gly-Asp) peptide-grafted oxidized sodium alginate hydrogel 3D culture system using a high-voltage electrostatic field. Compared to hydrogels containing gelatin, the proliferation rate of HepG2 cells in hydrogels containing collagen significantly increased starting from day 6, illustrating the proliferative capacity of collagen on tumor cells [116]. Although the above two 3D cell culture models have constructed TME-associated matrix components, it is challenging to cultivate in artificial or animal-derived environments that truly reflect the intratumoral environment of patients. A promising model recently is the decellularized tumor scaffold, which significantly preserves matrix components through decellularization of solid tumors. Comparisons with previously published healthy liver matrix profiles indicate that the overlap rate of matrix components can reach 96.4%. In the future, the application of decellularized tumor scaffolds in 3D cell culture models will provide more options for precision medicine research [117].

### Elastin

Elastin is a relatively stable matrix protein in the liver that participates in maintaining the structural integrity and function of the liver. The presence of elastin provides the liver tissue with necessary elasticity and toughness, enabling it to adapt to changes in blood flow and pressure. However, studies have found that elastin deposition is closely related to liver fibrosis, but unlike type I collagen, which almost disappears after the removal of damage signals, elastin significantly increases only in the late stages of liver fibrosis and still persists, which may be one reason why late-stage liver fibrosis is difficult to reverse [118]. Elastic fiber degradation products, specifically elastin-derived peptides (EDPs), are considered biomarkers of aging. They have been found to promote increased oxidative stress and inflammatory responses in the liver, which may accelerate the progression from Non-alcoholic fatty liver disease (NAFLD) to Non-alcoholic steatohepatitis (NASH). Research has also shown a correlation between EDPs and insulin resistance, making the inhibition of this target significant for the prevention of NAFLD-related liver cancer [23, 119]. Currently, there is a lack of research focusing on 3D cell culture models of elastin and liver cancer cells. Most studies utilize decellularization techniques which largely preserve various matrix proteins in the cellular microenvironment for subsequent biochemical experiments [120–122].

### Fibronectin

Fibronectin is an essential component of the extracellular matrix and is considered the primary organizer of the matrix [123]. Fibronectin is mainly classified into plasma fibronectin and cellular fibronectin, with plasma fibronectin synthesized by hepatocytes and existing in the blood as a soluble dimer. It is involved in biological processes such as liver injury repair and extracellular matrix assembly. However, in HCC research, it has been found that the oncogene MYC can promote the expression of fibrillin, thereby enhancing vascular infiltration in HCC, subsequent research has also confirmed the results [124]. Subsequent investigations have further validated these findings [24]. A recent study indicated that the contracted muscle in mice secretes fibrillin, activating hepatic autophagy, which induces insulin sensitization and improves systemic metabolic disorders [125]. However, the transcriptional upregulation of FN1 was not detected in the muscles of exercising humans, warranting cautious consideration regarding the applicability of these experimental results to human studies.

In 3D cell culture models, fibronectin is primarily considered as a cell adhesion substrate and its role in signal transduction with liver cancer cells. Research indicates that 6 kPa fibronectin microgels can significantly enhance the matrix remodeling and metabolic activity of HSC in mono- or multi-component microenvironments, providing new insights into in vitro remodeling of the TME [62]. One study also found that hepatocytes cultured on fibronectin-coated electrospun chitosan nanofiber scaffolds formed hepatocyte colonies while maintaining their morphology and function over time [126]. Building on this, Mansouri et al. generated fluorinated chitosan microparticles (MP) using a microfluidic chip, wrapping various liver ECM proteins, including fibronectin, in their periphery, and co-culturing with HepG2 and HSC, this model synergistically enhanced internal oxygen availability and the presentation of ECM cell adhesion ligands [127].

## Conclusions and future perspective

In HCC research, the transition from 2 to 3D in in vitro models signifies a deeper understanding of the disease among researchers. The onset of HCC is not merely due to the "seeds" of liver cells failing, but also involves alterations in the cultivated microenvironment or "soil," representing a shift in perspective from the individual to the whole. The TME of HCC continually evolves throughout its occurrence and progression, and the TME can vary significantly among different HCC patients. In today's context, to achieve personalized and precision medicine for patients, clinicians cannot overlook the TME. By constructing various in vitro 3D cell culture models targeting different components within the TME, we can explore the potential molecular mechanisms of interactions between liver cancer cells and microenvironment components.

**Fig. 3:**
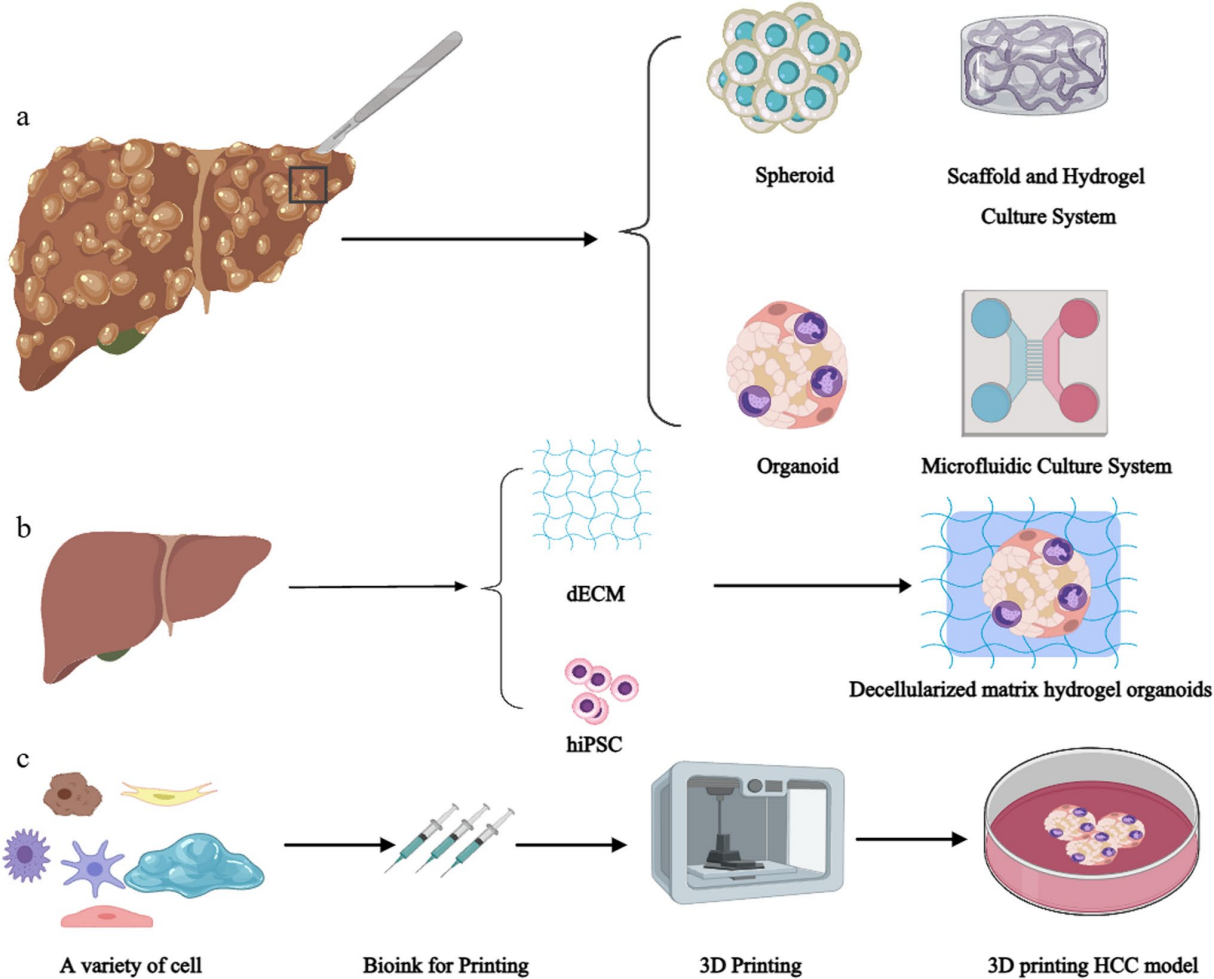
3D Culture Model Schematic. **a** Currently common hepatocellular carcinoma cell 3D culture models: tumor spheroids, organoids, 3D scaffolds, hydrogels, and microfluidic systems. **b** A model still under exploration: A 3D organoid model constructed using decellularized matrices derived from human liver tissue. Although it has not been fully realized yet, this model is anticipated to better reflect the liver cancer microenvironment and possesses good biocompatibility, retaining most of the matrix signals from the microenvironment. **c** A model that is not yet widely applied: An in vitro organoid model constructed using multiple cell types and mixed matrices as bio-ink through 3D printing technology. This model has the potential to preserve the heterogeneity of tumor cells and is expected to better simulate the tumor microenvironment, facilitating in-depth studies of tumor biology. Created with MedPeer (medpeer.cn)

Through reviewing and organizing relevant literature, we have found that tumor spheroid models are among the more commonly used models, possibly due to their simplicity and cost-effectiveness; however, they still fall short of accurately mimicking in vivo environments. Microfluidics and organoids have recently become popular areas of research, particularly organoids. Both models involve varying degrees of application of tissue engineering techniques. The primary advantage of organoids is their ability to replicate the microstructure and biological functions of in vivo organs, transforming the research subjects into "mini-organs" that can more realistically reflect the effects of external stimuli on in vivo tissues. Patient-derived cancer organoids can be established within a shorter time frame and are more cost-effective compared to patient-derived xenograft (PDX) models. In contrast, PDX models require large amounts of tissue samples, take up to six months to establish tumor growth, and often face complications from infiltrating murine stromal cells [128]. The development of liver cancer organoid models represents a highly promising alternative to animal models. By addressing ethical concerns and offering patient-specific and reproducible results, organoids align with the 3Rs principles (Replacement, Reduction, and Refinement), thereby providing a more ethical and translational approach to liver cancer research. While current organoid systems may not yet entirely replace all animal studies, continuous improvements and complementary applications have the potential to significantly reduce animal use and align research practices with evolving ethical standards. Although we have not yet identified studies focusing on immune checkpoints in liver cancer organoid models, PDOs cultured at the air–liquid interface have been shown to functionally recapitulate PD-1-dependent immune checkpoint activity in other cancers, such as non-small cell lung cancer, clear cell renal carcinoma, melanoma, and bladder urothelial carcinoma. For instance, in PDO models, treatment with anti-PD-1 drugs (e.g., nivolumab) significantly upregulated key effector molecules in tumor-infiltrating lymphocytes (TILs), such as IFNG, PRF1, and GZMB, and promoted the expansion of CD8^+^ T cells. Additionally, this model enables the evaluation of immunotherapy efficacy by analyzing functional biomarkers, such as TIL activation levels and tumor cytotoxicity [97]. Using PDO models to predict immunotherapy outcomes offers a promising alternative to traditional preclinical research tools. Despite current technical and systemic limitations, continuous innovations in PDO model engineering are expected to further unlock their full potential in immunotherapy research and drug development. Microfluidics constructs small chambers through bioengineering technology, allowing for the cultivation of cells within microfluidic environments; multiple microfluidic systems can directly exchange substances and information, offering high-throughput experimental capabilities. The 3D scaffolds and hydrogel culture systems mainly focus on extracellular matrix components from the TME, investigating the influence of non-cellular matrix elements on tumor cell proliferation, migration, and drug resistance by altering the viscosity and stiffness of the matrices. However, various 3D culture models are not mutually exclusive; the dissemination of scientific research has inspired diverse approaches, with some researchers integrating multiple models to derive novel, practical in vitro culture systems that align better with personalized medicine (Fig. 3).

The TME consists of cellular and acellular components, with interactions between cells and the influence of matrix signals on the cells occurring within the microenvironment. These components are also essential in in vitro studies. To simulate the in vivo environment, most in vitro models utilize co-culture with matrix cells. To introduce acellular components, researchers commonly employ decellularized tissue scaffolds as carriers. However, most studies tend to focus on specific factors, lacking a comprehensive and systematic approach (Table 1).

**Table 1:** Examples of 3D models for liver cancer in vitro

Models	Introduction	TME components	Advantages	Limitation	References
Spheroid	Spheroids are cell aggregates formed by the simple clustering of cells	Mainly consisting of tumor cell spheroids, lacking microenvironment components	• Considers interactions between tumor cells • Quick and easy to construct. Cost-effective and highly reproducible • More suitable for high-throughput screening and analysis	• Primarily represents the early stages of cancer • Differs significantly from the in vivo environment of patients	[48]
Organoid	A three-dimensional structure derived from (pluripotent) stem cells, progenitor cells, and/or differentiated cells that self-organizes through cell–cell and cell–matrix interactions, replicating various aspects of natural tissue structure and function in vitro	CAFs and their secreted matrix components	• Considering the interactions between tumor cells and CAFs	• Cholangiocarcinoma organoids, HCC organoids have not been constructed • Lack of involvement from other types of stromal cells	[44]
		HSCs, fibroblasts (WI38), and endothelial cells (HUVEC) and the extracellular matrix proteins they produce (such as collagen and fibronectin)	• Better simulates the in vivo environment • Particularly effective for studying drug-resistant tumors with high YAP/TAZ activity	• Model construction is relatively complex • Mixing tumor cells with stromal cells at a 1:1 ratio does not accurately reflect the in vivo extracellular matrix content	[45]
Microfluidics	By precisely controlling fluid flow within micron-scale channels, a suitable growth environment is provided for cells, promoting their growth, differentiation, and interactions	HSCs, endothelial cells, differentiated THP-1 derived Kupffer macrophages, COL I	• Requires minimal reagents and cell demands • Allows for quantitative assessment of vascularization, treatment effects, and gene expression • Enables high-throughput analysis and processing	• The model has limited adjustability	[53]
		Autologous peripheral blood mononuclear cells (PBMC) from healthy donors and allogeneic bone marrow-derived mesenchymal stem cells (BM-MSC)	• Provides an ideal, cost-effective platform • Lower drug screening costs and time. Higher efficiency and uniformity in organoid culture • Significantly reduces the time for high-throughput organoid culture and drug screening	• The impact of stromal components in the microenvironment has not been considered	[54]
Scaffold and hydrogel	Providing a three-dimensional scaffold or framework creates a growth environment for cells, better simulating the structure and function of in vivo biological tissues. Hydrogel materials can serve as a scaffold component, significantly enhancing the flexibility and functionality of cell culture by offering a physiologically relevant microenvironment	Hyaluronic acid	• Low cost • Enhanced proliferation ability of HepG2	• Lack of involvement of stromal cells • Significant difference from the in vivo environment	[55]
		HSCs、Fibronectin、COL I、COL III and COL IV	• Stiffness and protein are adjustable • Has high-throughput application potential	• The concentration of polyethylene glycol is still relatively high which may be detrimental to hepatocyte growth	[56]
		HUVEC、Hyaluronic acid	• Can be used for targeted vascular research、signal transduction and immune environment	• HUVEC are not sufficient to fully represent liver endothelial cells	[59]
		Gelatin and sodium alginate	• Preserved the genetic traits and expression profile of the original tumor • Achieved cell 3D bioprinting • Provided a foundation for subsequent multicellular 3D bioprinting	• Lack of involvement of other components in the microenvironment • The tissue sourced from patients requires at least 2.5 cm^3^ to ensure sufficient yield	[61]

Medical research does not rely solely on medicine; interdisciplinary collaboration is becoming a developmental trend that necessitates joint efforts from both medical professionals and engineers. In the process of constructing 3D cell models, researchers need to deal with numerous parameters and highly complex multidimensional data, including cell behavior, microenvironment characteristics, and drug responses. Machine learning and artificial intelligence (AI) tools can efficiently analyze and process these multidimensional datasets, extracting key regulatory factors and optimizing experimental design. However, the prerequisite for such applications is the availability of high-quality datasets to train the models. Rafieyan et al. utilized a dataset containing detailed information on 1171 scaffolds, which included data on various biomaterials and their concentrations (encompassing 60 natural and synthetic biomaterials, cross-linkers, and enzymes, among others), 49 cell lines, cell densities, and diverse printing conditions [129]. They applied over 40 machine learning and deep learning algorithms, combined with hyperparameter optimization, to uncover hidden patterns and predict outcomes such as cellular responses, printability, scaffold quality, and overall performance. This innovative approach not only significantly enhances research efficiency but also lays the groundwork for further applications of AI in tissue engineering, such as algorithm-based scaffold design. It holds great promise as a foundational milestone in this field. The combination of organoid co-culture technology with decellularized scaffold matrices demonstrates significant potential as a personalized preclinical model. Co-culturing patient-derived organoids with matrix cells effectively reflects the intercellular signals present in the patient's body. By cultivating on decellularized scaffold matrices derived from the patient's cancerous tissue, this approach maximally preserves the mechanical and chemical signaling of the matrix. Moreover, multi-cellular 3D bioprinted cancer organoids represent another promising model. However, the successful construction of these two models still faces numerous challenges, necessitating multidisciplinary collaboration to address the existing difficulties.

## Data Availability

No datasets were generated or analysed during the current study.
